# Characterization of the seed virome of alfalfa (*Medicago sativa* L)

**DOI:** 10.1186/s12985-023-02063-6

**Published:** 2023-05-19

**Authors:** Lev G. Nemchinov, Brian M. Irish, Sam Grinstead, Olga A. Postnikova

**Affiliations:** 1 Molecular Plant Pathology Laboratory, USDA-ARS, Beltsville, MD 20705 USA; 2 Plant Germplasm Introduction and Testing Research, USDA-ARS, Prosser, WA 99352 USA; 3 Animal Biosciences and Biotechnology Laboratory, USDA-ARS, Beltsville, MD 20705 US

**Keywords:** Alfalfa (*Medicago sativa* L.), Seed virome, Plant viruses, Seed transmission

## Abstract

**Background:**

Seed transmission of plant viruses can be important due to the role it plays in their dissemination to new areas and subsequent epidemics. Seed transmission largely depends on the ability of a virus to replicate in reproductive tissues and survive during the seed maturation process. It occurs through the infected embryo or mechanically through the contaminated seed coat. Alfalfa (*Medicago sativa* L.) is an important legume forage crop worldwide, and except for a few individual seedborne viruses infecting the crop, its seed virome is poorly known. The goal of this research was to perform initial seed screenings on alfalfa germplasm accessions maintained by the USDA ARS National Plant Germplasm System in order to identify pathogenic viruses and understand their potential for dissemination.

**Methods:**

For the detection of viruses, we used high throughput sequencing combined with bioinformatic tools and reverse transcription-polymerase chain reactions.

**Results:**

Our results suggest that, in addition to common viruses, alfalfa seeds are infected by other potentially pathogenic viral species that could be vertically transmitted to offspring.

**Conclusions:**

To the best of our knowledge, this is the first study of the alfalfa seed virome carried out by HTS technology. This initial screening of alfalfa germplasm accessions maintained by the NPGS showed that the crop’s mature seeds contain a broad range of viruses, some of which were not previously considered to be seed-transmitted. The information gathered will be used to update germplasm distribution policies and to make decisions on the safety of distributing germplasm based on viral presence.

**Supplementary Information:**

The online version contains supplementary material available at 10.1186/s12985-023-02063-6.

## Background

Plant viruses are transmitted from plant to plant by several principal modes: biological vectors, (arthropods, nematodes, and fungi); mechanical means via contaminated equipment, hands, or clothing; soil containing infected plant debris; vegetative propagation; and vertically, through seed and pollen [1]. Seed transmission, occurring mainly through the infected embryo, depends on the replication capacity of a virus in reproductive tissues and its survival during a seed maturation process [2]. Virus entry into the embryo can happen through direct invasion from the infected plant at different stages of organogenesis or via indirect routes such as fertilization with infected pollen [2, 3]. Even low rates of seed transmission can potentially result in long-distance dissemination of plant viruses and their subsequent introduction into the new areas [2, 4].

The current scope of knowledge on seed transmission of plant viruses in the agriculturally important forage crop alfalfa (*Medicago sativa* L.) is limited to a few individual viral species: alfalfa mosaic virus, and members of the families *Paritiviridae* and *Amalgaviridae.* Meanwhile, discovering the composition of the alfalfa seed virome and its implications for the distribution of pathogenic viruses to new territories has become increasingly important. Novel alfalfa viruses continue to be discovered at accelerated rates by high-throughput sequencing (HTS) technologies, demanding reevaluation of the impact of viral diseases on alfalfa health and of the plant’s role as a natural reservoir for dissemination of viruses to other agriculturally significant crops [5, 6].

The goal of this work was to perform initial seed screenings of alfalfa germplasm accessions maintained by the USDA ARS National Plant Germplasm System (NPGS) in order to identify potentially pathogenic viruses and evaluate their prospects for dissemination. The mission of the NPGS, among other activities, is to support agricultural production by distributing crop germplasm to ARS stakeholders, which often include plant breeders and scientists working in the field of alfalfa improvement. It is therefore critical to gather information on virus-free material and make appropriate decisions on whether germplasm distributions need to be restricted based on viral presence.

## Methods

### Plant material

Alfalfa seeds for ten different germplasm sources were acquired from the NPGS collection or from commercial sources (Table 1). Sample seed lots were chosen to be representative of diversity in the NPGS collection and included two commercially available cultivars.

**Table 1:** Descriptive information associated with alfalfa (*Medicago sativa* L.) germplasm seed samples evaluated in the current research

No.	Name	Identifier	Lot/inventory^1^	Description
1	BAM	PI 226470	90i	Landrace; acquired on 06/04/1955 from Iran
2	K30094	PI 346898	90i	Wild collected germplasm from Georgia; acquired on 12/24/1969
3	IG 131,609	PI 641657	2003i	Wild collected germplasm from Kyrgyzstan; acquired on 02/10/2002
4	ReGen	PI 643396	2019i	Modern breeding line; used in transformations; acquired in 2006
5	DON	PI 655519	2009o	Modern breeding line, diploid *M. sativa* subsp. *falcata*; acquired in 2008
6	Romagnoia	PI 672781	2004i	Landrace; acquired on 07/12/2000 from Italy
7	Maverick	W6 22304	Stdch3	Fall Dormancy (1) Standard Check cultivar (i.e., older cultivar)
8	CUF101	W6 22294	Stdch3	Fall Dormancy (9) Standard Check cultivar (i.e., older cultivar)
9	Commercial cultivar source 1*	N/A	N563-108 C	Modern commercial cultivar; OMRI^2^ coated
10	Commercial cultivar source 2*	N/A	N235-180 C	Modern commercial cultivar; OMRI^2^ coated

^1^For NPGS accessions with PI and W6 numbers, the inventory number is the year the current and available seed lot was last increased/regenerated. ^2^OMRI: Organic Materials Review Institute. *Commercial cultivar sources are coded omitting the identity of the genetic provider

### Total RNA extraction, RNA sequencing and RT-PCR

Prior to extraction, one gram of seed (~ 100 seeds) from each cultivar was surface-sterilized with concentrated sulfuric acid for one min then soaked in 70% ethanol for 1 min and rinsed with sterile water to eliminate all microorganisms including viruses, bacteria and fungi residing on the seed coat. Total RNA extraction was performed using Maxwell® RSC Plant RNA Kit according to the manufacturer’s directions (Promega Corp., Madison, WI USA). Psomagen (Psomagen Inc., Rockville MD USA) prepared cDNA libraries using Illumina TruSeq Stranded Total RNA Library Prep (Illumina Inc., San Diego, CA USA) and performed RNA-seq on a NovaSeq6000 S4 platform (150 bp, 1Gb, 20 million total reads, 10 M read pairs per sample). RNA extraction from germinated seedlings was done using RNeasy Plant Mini Kit (Qiagen Inc., Germantown, MD USA). Reverse transcription–polymerase chain reactions (RT-PCR) were performed using the SuperScript One-Step RT-PCR System according to the manufacturer’s directions (Thermo Fisher Scientific Inc., Waltham, MA USA). Primers specific for each tested virus were designed based on the results of the HTS and are shown in the Additional File 1. For control reactions, the SuperScript™ III RT/Platinum™ Taq Mix was substituted with Taq DNA Polymerase (Takara Bio USA Inc., San Jose, CA USA). The RT-PCRs were carried out in two technical replicas. The resultant amplicons were sequenced at the Psomagen facility (Psomagen Inc., Rockville, MD USA).

### Bioinformatic analysis

Sequence reads were trimmed using Trimmomatic, [7] then assembled with SPAdes [8]. The resulting contigs were screened using BLASTx searches [9] against a virus database containing all plant virus protein sequences from the NCBI RefSeq database (https://www.ncbi.nlm.nih.gov/refseq/). The resulting potential plant viral hits were searched once again using BLASTx against the full NCBI nr protein database. BBMap [10] was used to generate sequencing coverage values for the final hits.

## Results and discussion

**Fig. 1:**
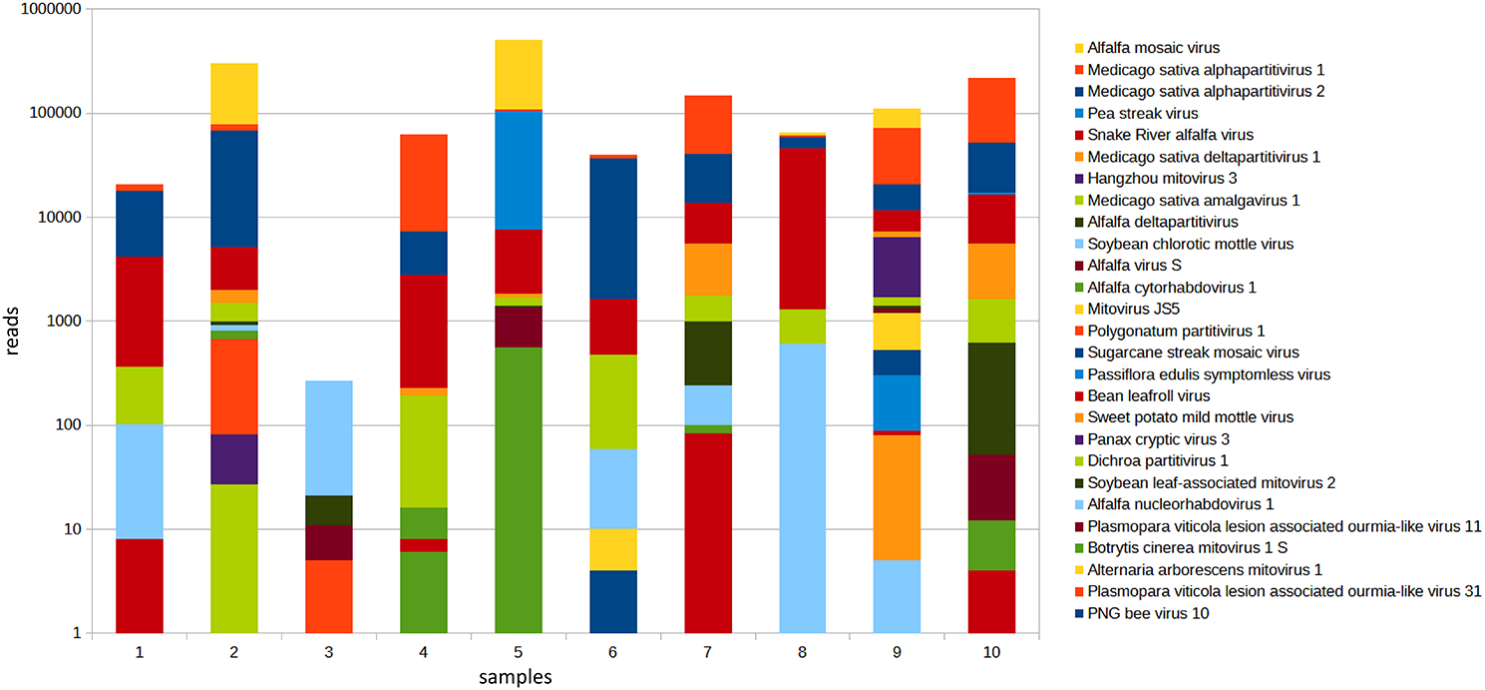
Distribution of viral communities in alfalfa (*Medicago sativa* L.) seed samples

In total, sequencing reads from 27 viruses were found collectively across all alfalfa seed germplasm sources (Table 2 and Additional File 2). Each of the seed samples averaged hits from 10 different viruses (Fig. 1). Based on the bioinformatic analysis, the viruses belong to no less than 15 genera representing 10 different families. Most prevalent among them were known seedborne and seed transmitted species such as alfalfa mosaic virus (AMV), Medicago sativa amalgavirus 1, and partitiviruses (Fig. 1). Their respective assembled contigs covered complete or near-complete genomes (Additional File 2). The identified members of the family *Partitiviridae* included unclassified viruses Panax cryptic virus 3 (46.1% protein identity), Dichroa partitivirus 1 (48.3%), and Polygonatum partitivirus 1 (69.5%) that have not been reported in alfalfa previously.

**Table 2:** A list of viruses identified by HTS in mature alfalfa (*Medicago sativa* L.) seeds of ten different germplasm sources

Virus names	Proposed taxonomy	Percent ID	E-value	Cultivar
Alfalfa cytorhabdovirus 2	Rhabdoviridae, Cytorhabdovirus	100	0	2,4,5,7,10
Alfalfa deltapartitivirus	Partitiviridae, Deltaparitivirus	100	0	2,5,7,9,10
Alfalfa mosaic virus	Bromoviridae, Alfamovirus	99.734	0	1,2,5–10
Alfalfa nucleorhabdovirus 1	Rhabdoviridae	98.086	2.55E-41	8,9
Alfalfa virus S	Alphaflexiviridae, Allexivirus	98.612	0	5,9,10
Alternaria arborescens mitovirus 1	Mitoviridae, Unuamitovirus	82.796	1.29E-41	6
Bean leafroll virus	Tombusviridae, Luteovirus	99.308	0	1,4,7,8,9,10
Botrytis cinerea mitovirus 1 S	Mitoviridae, Duamitovirus;	74.627	8.06E-25	4
Dichroa partitivirus 1	Partitiviridae, Unclassified	48.315	6.15E-44	2
Hangzhou mitovirus 3	Mitoviridae, Unclassified	31.052	8.30E-94	9
Medicago sativa alphapartitivirus 1	Partitiviridae, Alphapartitivirus	100	0	1,2,4–10
Medicago sativa alphapartitivirus 2	Partitiviridae, Aplphapartitivirus	100	0	1,2,4–10
Medicago sativa amalgavirus 1	Amalgaviridae, Unclassified	99.377	0	1,2,4–10
Medicago sativa deltapartitivirus 1	Partitiviridae, Deltapartitivirus	99.764	0	2,4,5,7,9,10
Mitovirus JS5	Mitoviridae, Mitovirus	31.068	1.46E-59	9
Panax cryptic virus 3	Partitiviridae, Unclassified	46.128	2.36E-79	2
Passiflora edulis symptomless virus	Potyviridae, Roymovirus	30.811	1.22E-127	9
Pea streak virus	Betaflexiviridae, Carlavirus	98.109	0	5, 10
Sweet potato mild mottle virus	Potyviridae, Ipomovirus	31.57	1.42E-59	9
Plasmopara viticola lesion associated ourmia-like virus 11	Mycovirus, Magoulivirus	68.539	6.34E-31	3
Plasmopara viticola lesion associated ourmia-like virus 31	Mycovirus, Ourmiavirus	94.667	4.96E-40	3
PNG bee virus 10	Iflaviridae, Unclassified	45.745	1.05E-14	6
Polygonatum partitivirus 1	Partitiviridae, Unclassified	69.535	0	2
Snake River alfalfa virus	Riboviria, Unclassified	100	0	1,2, 4–10
Soybean chlorotic mottle virus	Caulimoviridae, Soymovirus	65.924	0	1–3,5–9
Soybean leaf-associated mitovirus 2	Mitoviridae, Mitovirus	91.139	8.71E-38	3
Sugarcane streak mosaic virus	Potyviridae, Poacevirus	26.628	4.66E-83	9

Reads of bean leafroll virus (BLRV) and pea streak virus (PeSV), commonly infecting alfalfa, were also detected in several of the germplasm seed sources (Table 2). In some of them (germplasm sources 5 and 10), the assembled or overlapped contigs of the PeSV covered a near-complete viral genome (Additional File 2). As of today, both viruses are not considered seedborne although they have been previously found in alfalfa seeds [11]. While very few sequencing reads mapped to BLRV (~ 100 reads in all samples), nearly 100,000 of them aligned to the reference genome of PeSV (Additional File 2), indicating a likely seedborne nature of the virus, whether it is transmitted to offspring or merely localizes in the seed.

Alfalfa virus S (AVS), a member of the genus *Allexivirus*, was detected in the seeds of three different germplasm sources, including the two commercial cultivars. A near-complete AVS genome (~ 98.6% of GenBank ID: NC_034622.1) was recovered from the germplasm source 5 (Table 1, Additional File 2). We proposed earlier a potential role of seed transmission in the distribution of AVS [11]. The recently discovered Snake River alfalfa virus (SRAV) was also detected in the seeds of nine of the ten alfalfa germplasm samples. Some of them contained complete or nearly complete viral genome (germplasm sources 1, 2, 4, 5, 6, 7, 8, 9, and 10; Additional File 2). SRAV was proposed to belong to a flavi-like lineage [12] but later suggested to be a persistent, vertically transmitted virus distantly related to endornaviruses [13].

Several mitoviruses, for which the natural host is fungi, were most likely associated with fungal infections that can be carried internally in alfalfa seeds [14]. The exact fungal hosts of these mitoviruses are unclear, although suggestions can be made, contingent on the similarity scores with known viruses. The seed-infecting fungi would likely include economically important *Alternaria* spp., *Botrytis* spp., as well as *Peronospora* spp. in the Oomycota phylum.

Soybean chlorotic mottle virus (SbCMV) was recently reported to be an endogenous virus integrated into the alfalfa genome [15] and thus the presence of its genomic segments in the seed virome is not incidental. It is conceivable that the endogenous SbCMV-like elements are stable constituents of the host genome and have functional roles in alfalfa’s development. Whether they also represent a source of exogenous infection is currently unknown but cannot be excluded.

Scattered reads of two recently reported rhabdoviruses, alfalfa cytorhabdovirus (ACRV) and alfalfa nucleorhabdovirus (ANRV) [6] were found in the seeds of seven different germplasm sources. Larger genomic portions of these viruses may not have been recovered due to the possible limitations of RNA-seg depth. Rhabdoviruses are recognized as a cause of serious economic losses in plant crop species. A rhabdovirus infecting alfalfa in Argentina was associated with diseased plants displaying shortened internodes, a bushy appearance, deformations, puckering, epinasty of leaflet blades, vein enations, and varying sized papillae on the adaxial leaflet surfaces [16].

Several potyviral genomic fragments were recovered from commercial cultivar source 1. The longest (3.9 kb) translated contig had 26.6% identity with the polyprotein of sugarcane streak mosaic virus (SCSMV), covering the HC-Pro, P3, 6K1, and C1-encoding regions of the genome (PSI BLAST query cover = 79%; E-value = 3e-86), (Additional File 2). The second longest contig (3.1 kb) was 31.4% identical to the polyprotein of Passiflora edulis symptomless virus (PaeSV), covering 6K2, NIa-VPg, NIa-Pro, Peptidase C4, NIb and RdRp- encoding regions of the genome (PSI BLAST query cover = 99%; E-value = 3e-134). The third translated contig (1.3 kb) was 31.68% identical to sweet potato mild mottle virus (SPMMV), covering nearly complete coat protein of the virus (PSI BLAST query cover = 99%; E-value = 4e-55), respectively. It is thus possible that all these fragments represent a genome of one novel potyvirus, which we tentatively named alfalfa-associated potyvirus (AaPV1).

Seed transmission of potyviruses, which are among the most agriculturally significant plant viral pathogens, is not unusual, although its mechanism has not been completely described [17]. Research previously reported that pathogenic maize dwarf mosaic potyvirus (MDMV) was present in male and female floral organs at all organogenesis stages and was subsequently detected in mature pollen grains of the infected maize plants and all parts of the maturing seeds [3]. This suggests a systemic invasion of germ line by potyviruses via mother plant tissues. To our knowledge, no potyviruses have been detected or reported in alfalfa seed prior to this study. Traces of a virus distantly resembling PNG bee virus 10 [18] are likely incidental unless introduced by infected bees through pollen grains.

**Fig. 2:**
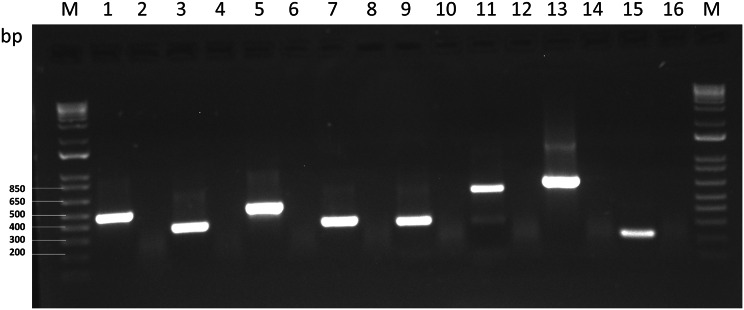
Reverse transcription-polymerase chain reaction to validate the presence of viral sequences in alfalfa (*Medicago sativa* L.) seeds. M, 1 kb Plus DNA ladder (Thermo Fisher Scientific Inc., Waltham, MA USA). Lane 1: amplification with primers LN1052/53 (BLRV, 473 bp), germplasm source №7. Lane 2: primers LN1052/53, control reaction. Lane 3: primers LN1054/55 (PeSV, 340 bp), germplasm source №5. Lane 4: primers LN1054/55, control reaction. Lane 5: primers LN1056/57 (ACRV, 480 bp), germplasm source № 5. Lane 6: primers LN1056/57, control reaction. Lane 7: primers LN1058/59 (AVS, 338 bp), germplasm source № 9. Lane 8: primers LN1058/59, control reaction. Lane 9: primers LN1060/61 (SRAV, 320 bp), germplasm source № 10. Lane 10: primers LN1060/61, control reaction. Lane 11: primers LN1062/63 (SCSMV, 603 bp), germplasm source № 9. Lane 12: primers LN1062/53, control reaction. Lane 13: primers LN1064/65 (PaeSV, 694 bp), germplasm source № 9. Lane 14: primers LN1064/65, control reaction. Lane 15: primers LN1066/67 (SPMMV, 213 bp), germplasm source № 9. Lane 16: primers LN1066/67, control reaction

In order to randomly confirm the presence of HTS-identified viruses and to exclude the possibilities of cross-contamination from other samples impacting the data, we performed RT-PCR with primers specific for several identified viruses: ACRV, AVS, BLRV, PeSV, SRAV, PaeSV, SPMMV, and SCSMV. Primers were designed based on the obtained HTS contigs (Additional File 1). The RT-PCR led to the amplification of the correct products from all these viruses (Fig. 2). This experiment validated the HTS findings. Nevertheless, as is always the case with HTS, the potential effect of contaminating sequences cannot be underestimated or ignored.

**Fig. 3:**
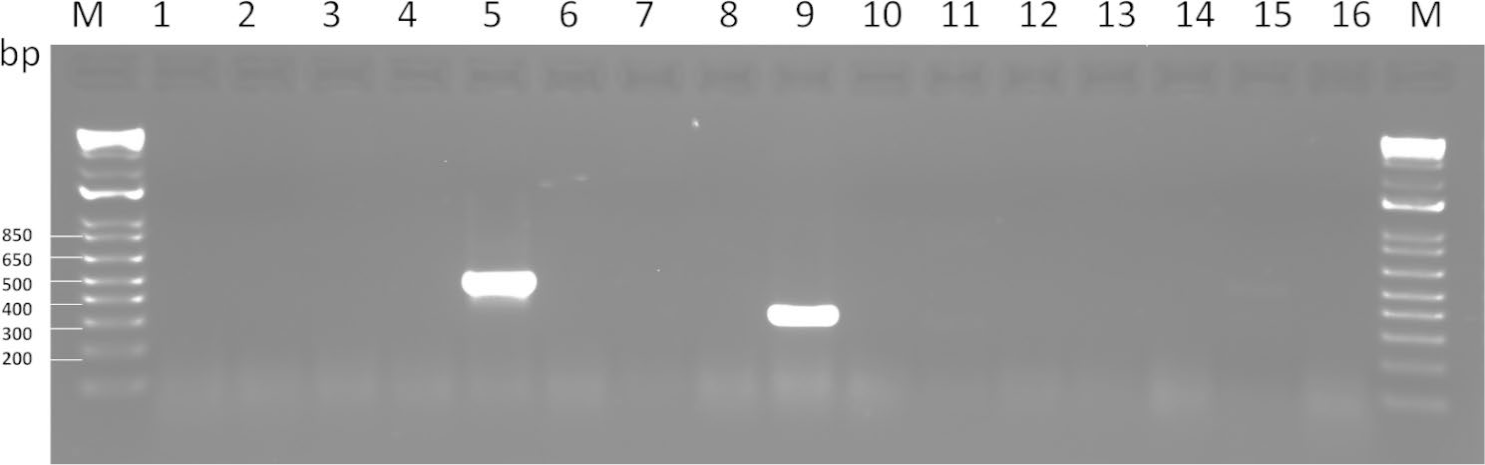
Reverse transcription-polymerase chain reaction validating seed transmission of the selected viruses in alfalfa (*Medicago sativa* L.). M, 1 kb Plus DNA ladder (Thermo Fisher Scientific Inc., Waltham, MA USA). Lane 1: amplification with primers LN1052/53 (BLRV), germplasm source №7. Lane 2: primers LN1052/53, control reaction. Lane 3: primers LN1054/55 (PeSV), germplasm source №5. Lane 4: primers LN1054/55, control reaction. Lane 5: primers LN1056/57 (ACRV, 480 bp), germplasm source № 5. Lane 6: primers LN1056/57, control reaction. Lane 7: primers LN1058/59 (AVS), germplasm source № 9. Lane 8: primers LN1058/59, control reaction. Lane 9: primers LN1060/61 (SRAV, 320 bp), germplasm source № 9. Lane 10: primers LN1060/61, control reaction. Lane 11: primers LN1062/63 (SCSMV), germplasm source № 9. Lane 12: primers LN1062/63, control reaction. Lane 13: primers LN1064/65 (PaeSV), germplasm source № 9. Lane 14: primers LN1064/65, control reaction. Lane 15: primers LN1066/67 (SPMMV), germplasm source № 10. Lane 16: primers LN1066/67, control reaction.

We next attempted to learn if the presence of the detected viruses in alfalfa seeds may lead to their actual seed transmission. For this purpose, we germinated surface-sterilized seeds of germplasm sources 5, 7, 8, 9, and 10 (Table 1) in Petri dishes. After one week, total RNA was extracted from seedlings and used for RT-PCR with the same sets of primers. The only amplicons produced were from ACRV and SRAV (Fig. 3). This experiment once again confirmed our previous suggestion of the persistent nature of SRAV in alfalfa [13]. The amplification of the ACRV sequence from germinated seedlings is of particular interest, since rhabdoviruses are not known to infect seeds of any plant species and mainly depend on transmission by phytophagous insects [19].

The remaining known and novel candidate viruses found in mature seeds by HTS and RT-PCR (AVS, BLRV, PeSV, PaeSV, SPMMV, and SCSMV) were likely unstable and inactivated in the embryo and thus did not retain their infectivity or were unable to replicate [2]. While seed transmission was not supported by this testing, it cannot be completely ruled out. It is also important to emphasize that all seeds used in this study, except for the commercial cultivars, have been maintained as accessions of the NPGS for a long period of time, some for as long as ~ 30 years, which could significantly affect virus transmissibility. From this perspective, it is remarkable that viral sequences were still detected in the seeds and some of them apparently retained infectivity.

## Conclusions

To the best of our knowledge, this is the first study of the alfalfa seed virome carried out by HTS technology. While a few individual seedborne viruses infecting the crop are well-known, the extent of the viral communities inhabiting seeds of this important forage legume was unexplored. Meanwhile, seed transmission can provide a source of primary infection for effective introduction into crops at an early age [1] or dispersal of a virus into new areas and subsequent viral disease epidemics [2]. It is also critical to point out that alfalfa could be a host reservoir for viruses causing significant losses in other crops [20]. This initial screening of alfalfa germplasm accessions maintained by the NPGS showed that the crop’s mature seeds contain a broad range of viruses, some of which were not previously considered to be seed-transmitted. The information gathered will be used to make decisions on whether germplasm distributions need to be scrutinized more carefully and in developing policies that restrict possible dissemination of confirmed plant pathogenic viruses. Follow up research might include a broader HTS-based survey of germplasm and/or commercial cultivars for viruses and into the possible effects these viruses have on crop production.

## Electronic supplementary material

Below is the link to the electronic supplementary material.

Additional File 1. Primers used in RT-PCR experiments.

Additional File 2. Viral contigs, descriptions, and sequences.

## Data Availability

All sequencing datasets mentioned in the text are included in the supplementary materials. The genomic sequences of the AaPV1 have been deposited in GenBank under the accession numbers OQ921098; OQ921099; and OQ921100

## Abbreviations

AVS: Alfalfa virus S
ACRV: Alfalfa cytorhabdovirus
ANRV: Alfalfa nucleorhabdovirus
BLRV: Bean leafroll virus
HTS: High-throughput sequencing
MDMV: Maize dwarf mosaic potyvirus
NPGS: National Plant Germplasm System
PaeSC: Passiflora edulis symptomless virus
PeSV: Pea streak virus
SbCMV: Soybean chlorotic mottle virus
SRAV: Snake River alfalfa virus
SCSMV: Sugarcane streak mosaic virus
SPMMV: Sweet potato mild mottle virus
RdRp: RNA-dependent RNA polymerase
RACE: Rapid Amplification of cDNA Ends
RT-PCR: Reverse transcription-polymerase chain reaction
